# Supplementary material for: Probing multiphoton light-induced molecular potentials
Source: Nat Commun. 2020 May 22;11:2596. doi: 10.1038/s41467-020-16422-2 (PMC7244592; doi:10.1038/s41467-020-16422-2)
Supplement: Supplementary file 1 — Supplementary Information [file 41467_2020_16422_MOESM1_ESM.pdf]

Supplementary information to

Probing multiphoton light-induced molecular potentials

M. Kübel, M. Spanner, Z. Dube, A. Naumov, S. Chelkowski, A.D. Bandrauk, M.J.J. Vrakking, P.B. Corkum, D.M. Villeneuve, A. Staudte

Supplementary Note 1: Bond-softening in mid-IR fields of different intensities

The discussion of Figure 1 of the main text argues that multiphoton couplings become increasingly important at long wavelengths. In Supplementary Figure 1, we plot the calculated Floquet states for H_2^+ dressed by 2300 nm light of various intensities. As the intensity increases, new multiphoton pathways open up, as indicated in the figure. Notably, the one-photon crossing is already wide open at a very moderate intensity of 10^{11} Wcm^{-2}. Three-photon coupling becomes significant already in the low 10^{12} Wcm^{-2} range. This contrasts with the visible regime, where approximately ten times higher intensity is required for the three-photon coupling to become significant.

As further shown in Supplementary Figure 1, an intensity of 10^{13} Wcm^{-2} as used in the present experiment is sufficient for the 5-photon crossing to open up. Notably, ionization of neutral H_2 requires intensities of the order of 10^{14} Wcm^{-2}, when couplings of orders 7 and 9 and higher become relevant.

The curvatures of the energy surfaces around the multiphoton crossings are rather unique. Conical intersections are characterized as points of degeneracy between two electronic potential energy surfaces where the degeneracy is lifted to first order, i.e., linearly, for small displacements of the nuclear coordinates around this point of degeneracy along at least two directions in the coordinate space[1]. Non-conical intersections, for example, Renner-Teller intersections, do not share this property. In the case of Renner-Teller intersections, the degeneracy is lifted to second order, i.e.,

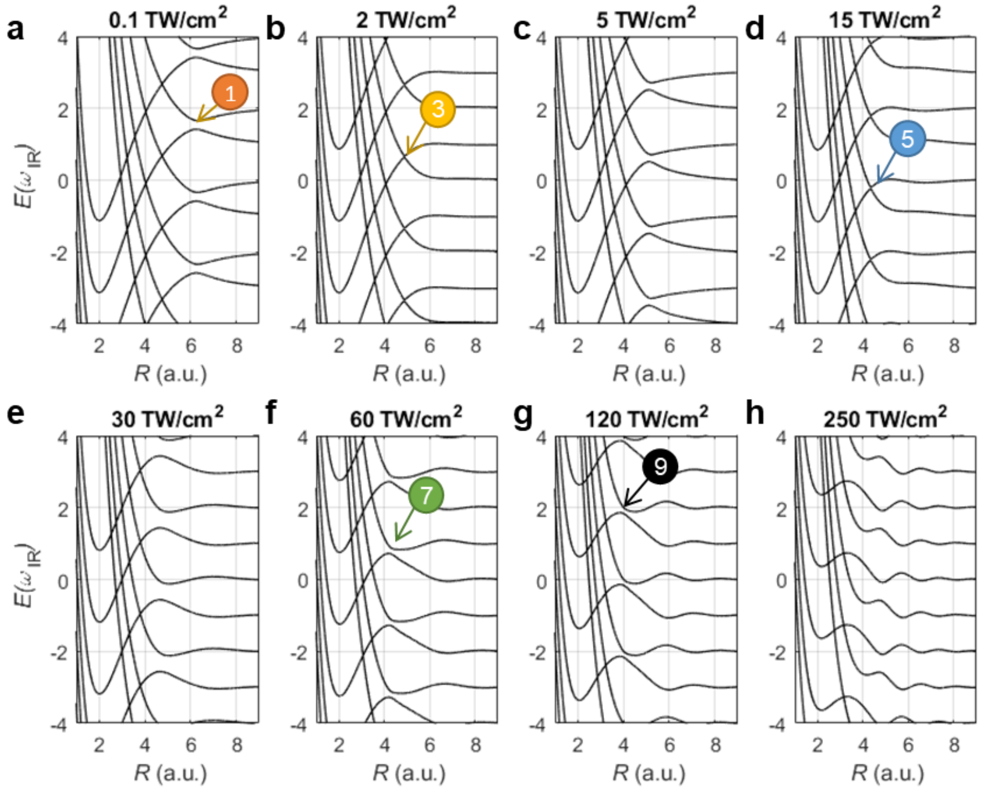

Supplementary Figure 1. Mid-IR light-induced potentials in H_2^+. Laser-dressed potential energy curves have been calculated for various intensity values given in the panel titles and a wavelength of 2300 nm. The numbers in the circles indicate dissociation channels that open up as n-photon crossings turn into avoided crossings with increasing intensity.

quadratically, as the nuclear coordinates are perturbed away from the degenerate point [1]. In the case of the one-photon crossing, i.e., the LICI, the degeneracy is lifted linearly for small displacements along both the radial and angular coordinates, just like a standard field-free conical intersection. However, the multiphoton crossings behave differently. While the degeneracies are lifted linearly along the radial coordinate for all of the multiphoton crossings, this is no longer the case along the angular coordinate. For the 3-photon crossing the degeneracy is lifted to the third order for small displacements along the angular coordinate, and for the 5-photon crossing the degeneracy is lifted to the fifth order, see Supplementary Figure 2. In general, the degeneracy is lifted to the *n*-th power for small angular displacements for the *n*-photon crossing. The multiphoton crossings therefore lift the degeneracy linearly for small displacements along only one direction (the radial direction) and nonlinearly along the other (the angular direction).

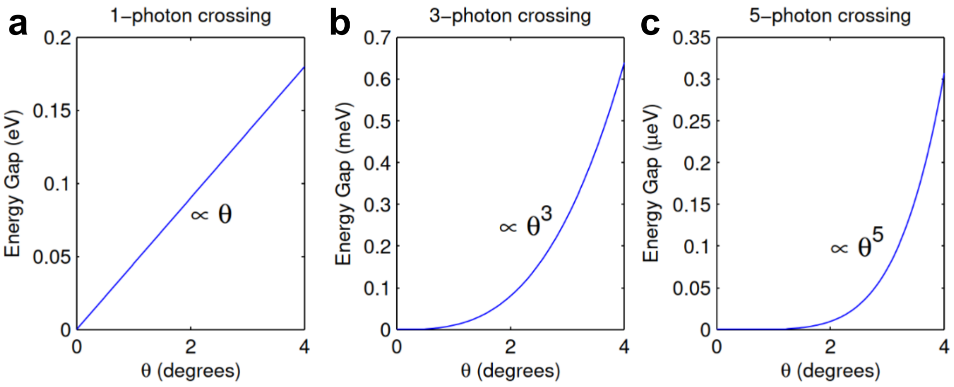

Supplementary Figure 2. Linear and non-linear avoided crossings. Energy gaps between the crossing surfaces as a function of small angular displacements along the θ coordinate, demonstrating how the degeneracies of the point intersections are lifted to different orders for the single and multiphoton crossings. For these plots, the IR field intensity was 4x10^{14} W/cm^2.

We explore the different intensity regimes numerically by solving the time dependent Schrödinger equation (TDSE) for various field parameters. In Supplementary Figure 3, we present proton momentum distributions calculated for different intensity regimes, and separated by the electronic state on which the molecular ion dissociates. At low intensity, the signal is dominated by single photon dissociation leading to a smooth angular distribution. At higher intensities, various multiphoton pathways contribute to the proton yield and lead to structured angular distributions.

However, the distributions also become more confined along the laser polarization due to rotational alignment of the molecular ion prior to breakup. While there is little angular structure in the momentum distributions produced at low intensities, the rich and convoluted structures obtained at high intensity are unlikely to be resolvable in an experiment. This suggests that the intermediate intensity regime in the low 10^{13} Wcm^{-2} range is the most promising regime to selectively probe multi-photon effects in the light-induced potential energy landscape.

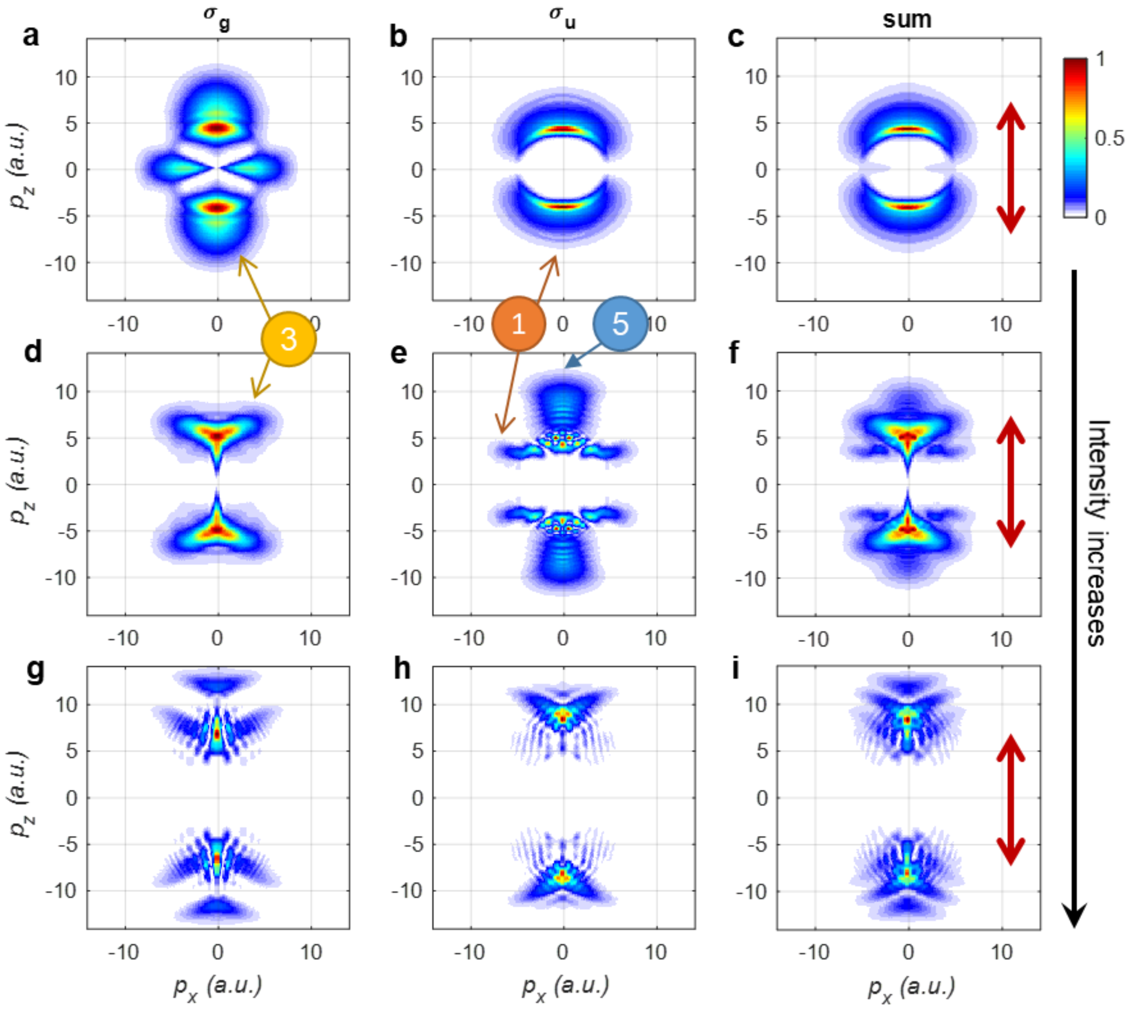

Supplementary Figure 3. Bond softening regimes for mid-IR driving pulses. Proton momentum distributions obtained from TDSE calculations for mid-IR (2300 nm, 50 fs) laser pulses of various intensities, (a-c) the single-photon regime, 5×10^{12} Wcm^{-2}, (d-f) the multi-photon regime, 3×10^{13} Wcm^{-2}, and (g-i) the ionization regime, 1.8×10^{14} Wcm^{-2}. Signals arising from various n-photon couplings are indicated in panels (a,b) and (d,e). Such an assignment is no longer unambiguous in (g-i), where contributions from different multiphoton orders overlap due to rotational alignment along the laser polarization. For each intensity, the signals dissociating on the σ_g (a, d, g) and σ_u (b,e,h) surfaces, as well as their sum (c,f,i) are shown. Each panel is normalized to its maximum. The red arrows indicate the laser polarization.

Supplementary Note 2: Two-color Floquet results

Floquet theory requires the Hamiltonian to be periodic in time. While the few-cycle pulse used for ionization exhibit only little periodicity, it is well known that few-cycle pulses often exhibit a pulse pedestal, which is much longer than the full width at half maximum pulse duration[2], and can play a significant role in H_2 fragmentation[3,4]. Comparing the experimental results using the visible pulse alone (Figure 2(b) of the main text) to simulations, suggests that an intensity of approximately $1\text{x}10^{13}$ Wcm^{-2} is appropriate for calculating the effect of the visible pulse on the field dressed states. To obtain a periodic two-field, we further take the frequency of the visible field as 3 times the frequency of the mid-IR. This is a good approximation, as the carrier wavelength of the visible pulse (730 nm) is almost exactly 1/3 of the 2300 nm mid-IR wavelength. For the calculation of the Floquet states at each molecular alignment angle θ, the effective laser field is calculated according to

$$F(\theta) = F_{\text{VIS}}(\theta) + F_{\text{IR}}(\theta)$$

$$F_{\text{VIS}}(\theta) = F_{0,\text{VIS}}\cos(\theta)$$

$$F_{\text{IR}}(\theta) = F_{0,\text{IR}}\sin(\theta) \qquad \textbf{(1)}$$

where θ is the molecular alignment angle in the polarization plane, as indicated in Supplementary Figure 4, and $F_{0,\text{VIS}}$ / $F_{0,\text{IR}}$ are the field strengths of visible and IR pulses, respectively.

Supplementary Figure 4 (a) reproduce the LIP landscape presented in Figure 3(d) of the main text, and Supplementary Figure 4 (b,c) shows the TDSE results presented in Figure 3(a,b) of the main text. Below we discuss various structures in the LIP landscape on the basis of the two-colour Floquet states at fixed angles presented in Supplementary Figure 4 (d-i).

Beginning at $\theta = 0°$, Supplementary Fig. 4(d), the visible pulse couples the σ_g and σ_u states with 1 photon, opening a gap at R≈4.7 a.u., which coincides with the 3-photon crossing of the IR field. The 1-photon crossing of the IR field is closed, leading to dissociation towards the $-3\omega_{\text{IR}}$ dissociation limit along the polarization axis of the visible pulse.

At slightly larger θ, Supplementary Fig. 4(e), the one-photon crossing of the IR opens up, inhibiting dissociation towards the $-3\omega_{IR}$ as for $\theta \approx 0°$. Instead, the molecule can only dissociate towards the $-2\omega_{IR}$ limit. Thus, the dissociation momentum of the proton emitted around 10° is reduced with respect to the dissociation at $\theta = 0°$.

The 1-photon crossing continues to open through $\theta = 20°$. At $\theta \approx 30°$, Supplementary Fig. 4(f), the width of the 1-photon crossing reaches $2\omega_{IR}$, thus closing the gap with the state dissociating towards the $-2\omega_{IR}$ limit. This leads to the pronounced LICI between the yellow and red surfaces in Supplementary Fig. 4(a). As the comparison of Supplementary Fig. 4(b) and (c), shows, rotational dynamics lead to redistribution of the fragments to other angles, where dissociation is favoured. This rotational redistribution is indicated by the black arrows in Supplementary Figs 4(b) and (c). The minimum in the proton signal at $\theta \approx 30°$ is a clear signature of the LICI.

At $\theta \approx 40°$ (Supplementary Fig. 4(f)), the 3-photon gap of the IR field is wide open, again allowing transition to the $-3\omega_{IR}$ limit. Here, a clear maximum in the fragment yield is obtained, especially in the results including rotational dynamics.

At larger angles $\theta \approx 60°$, the gap at $R = 4$ *a.u.* nearly closes, which is attributed to the 5-photon coupling by the IR field. This suppresses dissociation. At $\theta \approx 90°$, the 5-photon dissociation channel dominates the dissociation process.

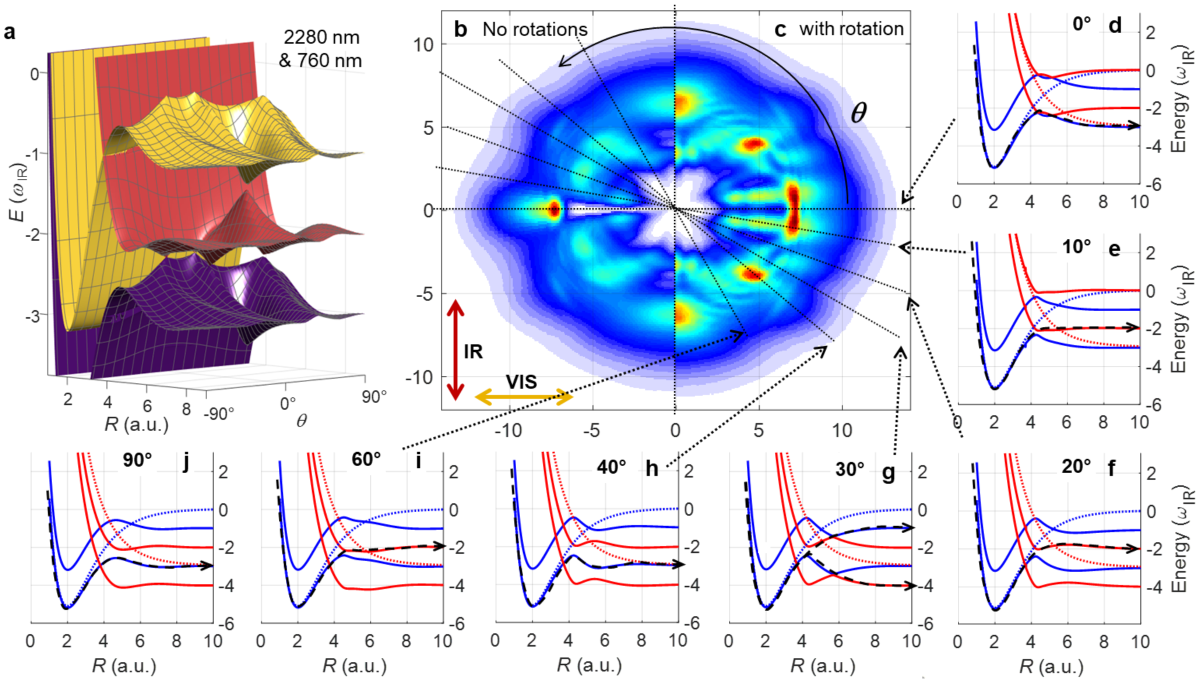

Supplementary Figure 4. Angle-dependent channel switching. (a) Light dressed states in an ω-3ω two-color laser fields (767 nm, 5×10^{12} Wcm^{-2}; and 2300 nm, 3×10^{13} Wcm^{-2}) where the two-color phase is 0. TDSE results for the proton momentum distribution (b) without and (c) with rotational dynamics. The orange arrows indicate population transfer through molecular rotation. (d-j) Selected two-color Floquet states of H_2^+ calculated for various molecular alignment angles, as indicated. Red and blue colors indicate states of opposite parity. The dotted lines show the undistorted σ_g (blue) and σ_g -3ω_{IR} (red) states. The dashed black arrows indicate a possible dissociation pathway.

Supplementary Note 3: Streaking Measurement of the mid-infrared pulse

The mid-IR pulse in our experiment deflects the photoelectrons liberated around the field maxima of the visible pulse. The deflection corresponds to the instantaneous vector potential of the mid-IR field at the time of ionization[5]. Thus, the delay dependent momentum distribution along the mid-IR polarization, called STIER trace[6], images the mid-IR vector potential as a function of time.

In Supplementary Figure 5, we present the STIER traces for the H_2 and D_2 experiments for which data is presented in the main text. The laser parameters extracted from the traces are as follows:

- *Peak Intensity* I_{IR} = (3.3 ± 0.3) × 10^{13} Wcm^{-2}
- *Pulse duration (FWHM intensity):* τ = (45 ± 2) fs
- λ *= 2336 nm*

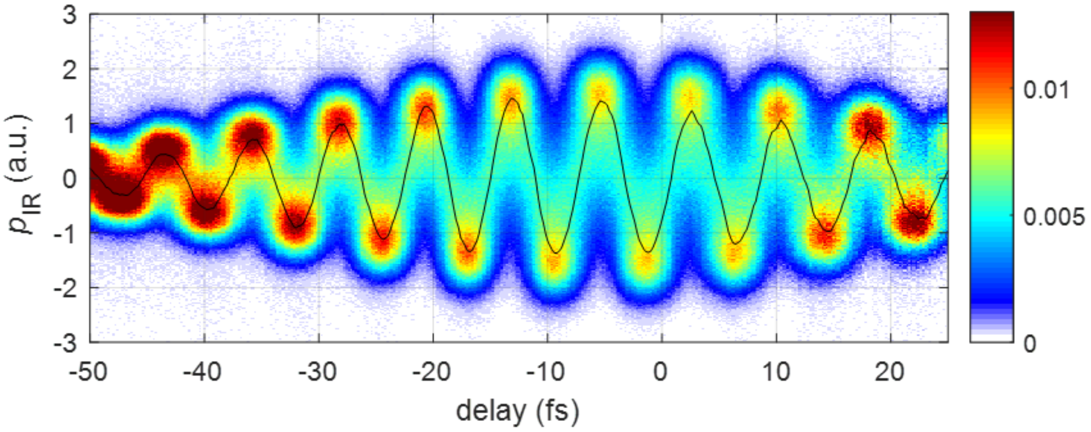

Supplementary Figure 5. Streaking of strong-field ionization using STIER. Shown is the delay-dependent H_2^+ momentum distribution along the mid-IR polarization. The solid line indicates the delay-dependent shift of the centroid of the momentum distribution. It constitutes an estimate of the vector potential of the phase-stable mid-IR pulse.

Supplementary Note 4: Effect of the electron recoil momentum

In our experiment, neutral H_2 is dissociatively ionized by the intense laser field, producing three particles, a neutral H atom, an H^+ ion, and an electron. The momentum balance in the laboratory frame reads (neglecting the very small photon momenta),

$$\mathbf{p}_H^L + \mathbf{p}_i^L + \mathbf{p}_e^L = 0, \qquad (2)$$

Here, $\mathbf{p}_i^L$ and $\mathbf{p}_e^L$ are the ion and electron momenta in the laboratory frame, that are measured in coincidence by the COLTRIMS apparatus, and $\mathbf{p}_H^L$ is the momentum of the neutral that is not recorded. As we are interested in probing the molecular dynamics in the H_2^+ ion, we transform to the recoil frame by calculating

$$\mathbf{p}_i^M = \mathbf{p}_i^L + \mathbf{p}_e^L / 2 \qquad \text{and} \qquad \mathbf{p}_H^M = \mathbf{p}_i^L + \mathbf{p}_e^L / 2. \qquad (3)$$

Hence, in the recoil frame, the momentum balance for the dissociation reads

$$(1)\ \ \mathbf{p}_H^M + \mathbf{p}_i^M = 0, \qquad (4)$$

and $\mathbf{p}_H^M$ originates in the dissociation process only, thus probing the LIPs. Note that equation (3) assumes that dissociation occurs on a time scale longer than the pulse duration. Thus, in practice, the transformation into the recoil frame removes the influence of the electron recoil only partially.

In Supplementary Figure 6 (a,b), we compare recoil-frame and laboratory-frame momentum distributions for H^+. The comparison to the TDSE results in Supplementary Figure 6(c), shows that the sharp structures visible in the recoil-frame are consistent with those obtained in the computational results.

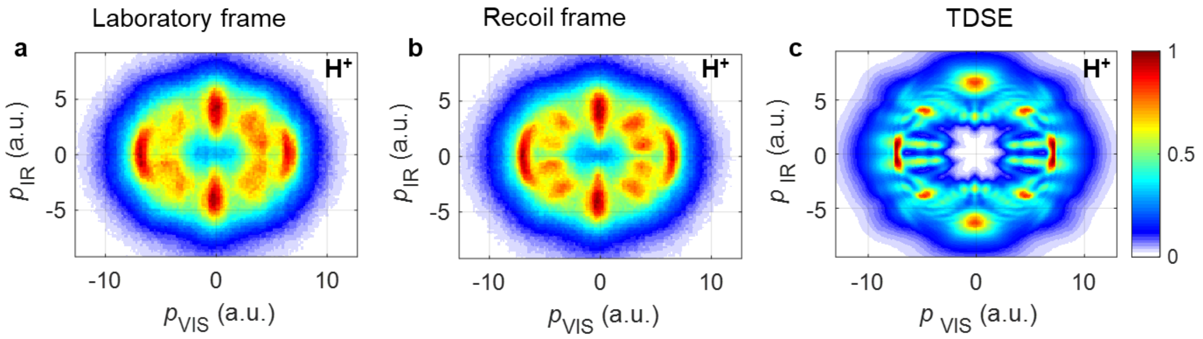

Supplementary Figure 6. Laboratory and recoil frame measurements. Panels (a,b) show the same data obtained from H_2, but presented in (a) the laboratory frame and (b) the recoil frame. They differ by the half the photoelectron momentum, which was recorded in coincidence with the protons. TDSE results for two-colour bond softening of H_2^+ are shown in (c).

Supplementary Note 5: Delay-dependence of the TDSE results

TDSE Simulations for the proton momentum distribution at different time delays are presented in Supplementary Figure 7. These results should be compared to the Figure 4 of the main text.

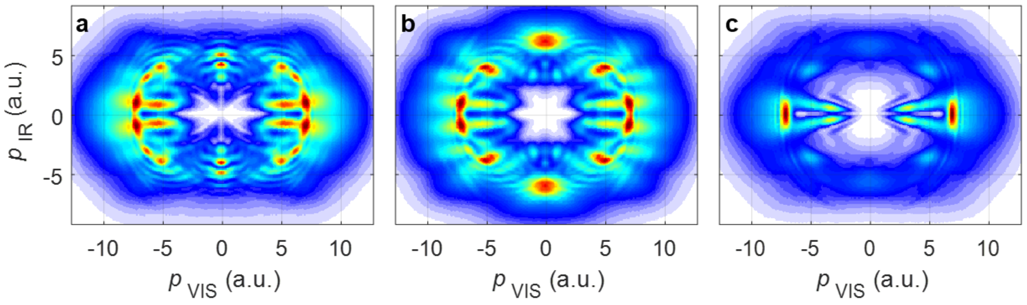

Supplementary Figure 7. Simulated proton momentum distributions for different time delays. The spectra were calculated for temporal overlap of the visible pulse with the (a) rising edge (Δt = -30fs), (b) the center (Δt = -10fs), and (c) the falling edge (Δt = +10fs) of the 45 fs mid-IR pulse. The spectra were integrated over a delay range of ±2 fs and over the CEP of the visible pulse.

Supplementary Note 6: Transition from single to two color dressing fields

In Supplementary Figure 8, we present the results of our TDSE simulation in which the pedestal of the visible pulse has been removed. Supplementary Figure 8 (a) reproduces the results show in Figure 1 (e) of the manuscript. Supplementary Figures 8 (b) and (c) show the proton momentum distributions obtained with the IR pulse and a clean few-cycle visible pulse of two different pulse durations. Evidently, the dissociation dynamics is affected by a 5-fs pulse, while a 7-fs pulse already produces angular dissociation patterns that are reminiscent of our experimental results. This shows that probing the LIPs resulting from the IR field only, in fact, requires extremely short and clean pulses.

Even without such pulses, however, the reasons for the access to high-order point-intersections, i.e., the relatively low intensity and long wavelength, continue to remain operative even in the presence of a second, perpendicularly polarized light field as the structural progression in the proton spectra in Supplementary Figure 8 clearly shows. Thus, the nuclear wavepacket propagates on the combined two-color high-order potential energy surfaces shown in Fig. 3c.

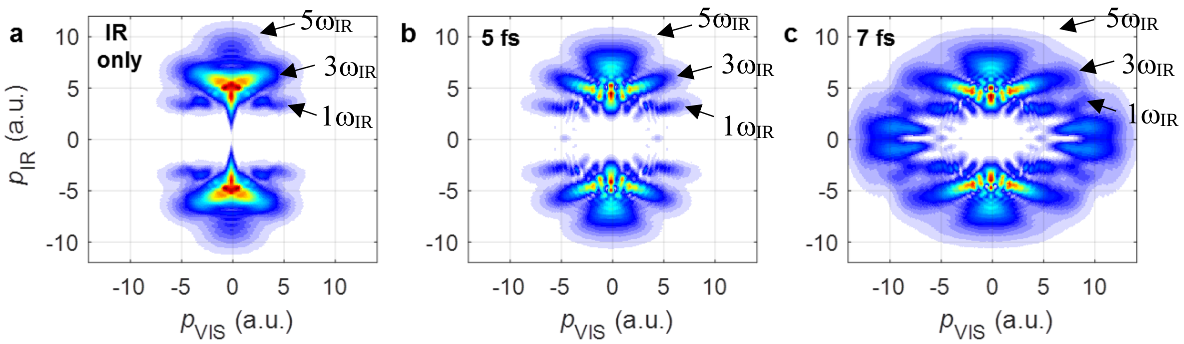

Supplementary Figure 8 Transition from one-color to two-color dissociation. Calculated proton momentum distribution arising from the photodissociation of H_2^+ by a 45-fs mid-IR (2300 nm) pulse at $3 \cdot 10^{13}$Wcm^{-2} (a) only, (b) in combination with a 5-fs visible pulse, (c) in combination with a 7-fs visible pulse. The visible pulse has a carrier wavelength of 730 nm and a peak intensity of $2 \cdot 10^{14}$Wcm^{-2}.

Supplementary Note 7: Delay dependence for single-color dissociation

In order to emphasize the impoWrtance of using a two-pulse scheme, we present additional computational results in Supplementary Figure 9 for two cases. In the first case (a-d) the simulation is initialized in the center of the mid-IR pulse. This is the situation for which the results shown in Fig. 1(e) of the manuscript were obtained. In the second case (e-h), the simulation is initialized at the onset of the mid-IR pulse. This situation is similar (but not identical) to performing the experiment with in ion beam apparatus that produces H_2^+ ions.

As can be seen in Supplementary Figure 9, the results for the two cases are quite different. In particular, in Supplementary Figure 9(h), a very convoluted pattern is produced that is strongly aligned along the laser polarization, such that non-linear features cannot be unambiguously identified. This comparison emphasizes the importance of the pump-probe scheme in order to study the effect of multiphoton light induced potentials.

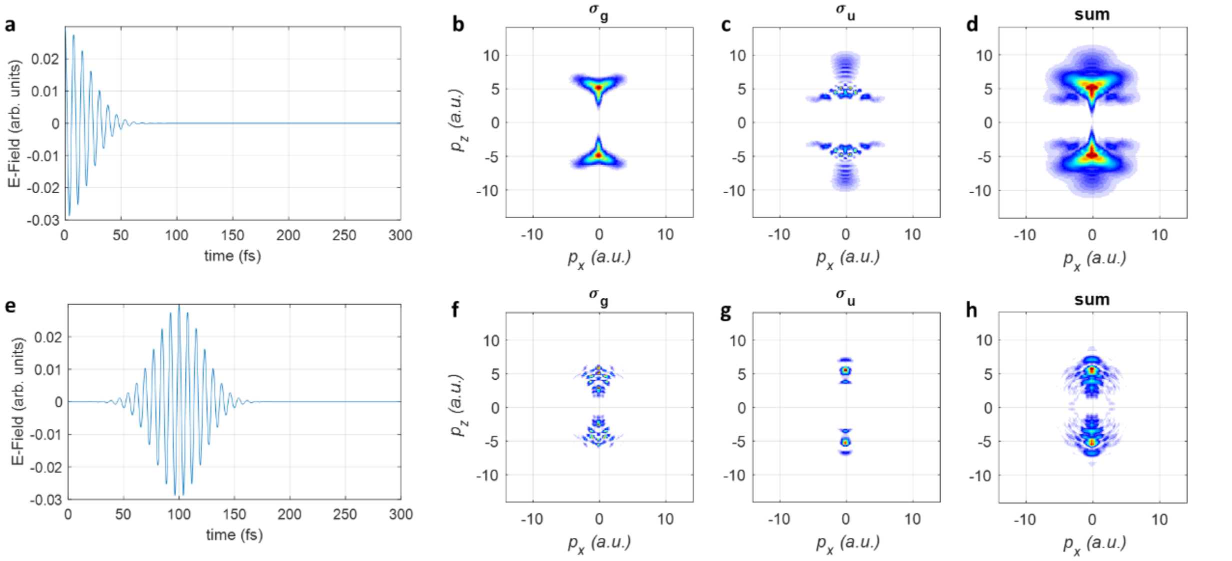

Supplementary Figure 9. Effect of the pump probe scheme for separating multiphoton contributions. Dissociation of H_2^+ when the molecular ion is created (a-d) in the center or (e-h) at the onset of a mid-IR pulse (35 fs, $3 \cdot 10^{13}$ W/cm², 2300 nm). Shown are (a,e) the electric field experienced by the molecular ion, and the proton momentum distribution for molecules dissociating on the σ_g (b, f, respectively) or σ_u (c, g, respectively) surface, as well as (d, h, respectively) the total dissociation signal.

Supplementary References

1. Domcke, W., Yarkony, D. R. & Köppel, H. *Conical intersections: theory, computation and experiment*. vol. 17 (World Scientific, 2011).
2. Kim, K. T. *et al.* Petahertz optical oscilloscope. *Nat. Photonics* **7**, 958 (2013).
3. Xu, H., He, F., Kielpinski, D., Sang, R. T. & Litvinyuk, I. V. Experimental observation of the elusive double-peak structure in R-dependent strong-field ionization rate of H_2^+. *Sci. Rep.* **5**, 13527 (2015).
4. Li, H. *et al.* Intensity dependence of the attosecond control of the dissociative ionization of D_2. *J. Phys. B At. Mol. Opt. Phys.* **47**, 124020 (2014).
5. Itatani, J. *et al.* Attosecond Streak Camera. *Phys. Rev. Lett.* **88**, 173903 (2002).
6. Kübel, M. *et al.* Streak Camera for Strong-Field Ionization. *Phys. Rev. Lett.* **119**, 183201 (2017).
